# Research trends of platelet-rich plasma therapy on knee osteoarthritis from 2011 to 2021: A review

**DOI:** 10.1097/MD.0000000000032434

**Published:** 2023-01-13

**Authors:** Yubo Cui, Liqiong Lin, Zhiwei Wang, Kai Wang, Lili Xiao, Wentao Lin, Yiyuan Zhang

**Affiliations:** a Department of Orthopedics, Fuzhou Second Hospital Affiliated to Xiamen University, Fuzhou, Fujian, China; b The Second Clinical Medical College of Fujian University of Traditional Chinese Medicine, Fuzhou, Fujian, China; c Medical College of Xiamen University, Xiamen, Fujian, China.

**Keywords:** bibliometric analysis, citespace, knee osteoarthritis, platelet-rich plasma

## Abstract

Platelet-rich plasma (PRP) has been widely used to alleviate osteoarthritis of the knee, and research results are abundant. However, there are no bibliometric reports in publications in this field. Therefore, the current status of PRP for the treatment of osteoarthritis of the knee from 2011 to 2021 was analyzed using Citespace 6.1.R2 software.

**Methods:**

Publications regarding PRP treating Knee Osteoarthritis between 2011 and 2021 were extracted from the Web of Science database. CiteSpace was used to analyze the number of publications, countries, institutions, journals, authors, cited references, and keywords by using standard bibliometric indicators.

**Results:**

A total of 988 publications were searched from 2011 to 2021. In the last decade, the number of publications has increased in the field. Brian J. Cole was the author with the most output, with 31 relevant articles, and Giuseppe Filardo ranked first in cited authors. *Am J Sport Med* was the most cited journal. In this field, the most prolific country is the United States and the most prolific institution is Rush University. An article published by Sandeep Patel ranked first in cited references with 118 citations. “Randomized controlled trial” was the most bursting keyword and other more popular keywords about PRP for knee osteoarthritis: “hyaluronic acid,” “double-blind,” and “mesenchymal stem cell.”

**Conclusion:**

This bibliometric study provides a decade of current clinical research on PRP for the treatment of osteoarthritis of the knee, which can help researchers understand the hot spots in the field and provide a new direction for their research.

## 1. Introduction

Knee osteoarthritis (KOA) is a severe degenerative disease that results in cartilage loss due to mechanical loading, affecting all tissues within the joint (e.g., synovium, ligaments) and leading to significant changes in tissue structure, metabolism, and function.^[1,2]^ It causes pain and limited knee motion, which greatly reduces the quality of life of patients.^[3]^ The prevalence of KOA has increased significantly in the last decades. The prevalence of KOA in adults Chinese over 60 years of age is estimated to be about 10% in men and 13% in women.^[1,4]^ A combination of therapeutic modalities including non-pharmacological and pharmacological interventions remains the key to the treatment of KOA, with treatment being mainly by weight control, exercise, and pharmacological treatments such as non-steroidal anti-inflammatory drugs, glucosamine, chondroitin, or intra-articular corticosteroid injections.^[5,6]^ Patients intervene in disease progression with various treatment modalities, but with the exception of joint replacement surgery, individualized differences in treatment outcomes are large depending on the severity of KOA.^[7,8]^ Arthroplasty is an effective definitive treatment for advanced KOA but is not indicated for non-end-stage disease and younger patients. it is expensive and carries the risk of serious postoperative complications.^[9,10]^ Therefore, to find a more effective treatment, more and more studies are focusing on injecting platelet-rich plasma (PRP) into the diseased knee joint. PRP is an autologous blood product in which a small amount of peripheral blood is obtained aseptically and centrifuged to concentrate platelets in plasma, the concentration of which depends on different centrifugation equipment, gravity, and centrifugation time as well as on the individual patient.^[11–14]^Platelets *α*-granules (e.g., TGF-*β*1, PDGF, bFGF, VEGF, EGF, IGF-1) contain large amounts of growth factors, which are concentrated and injected into the knee joint to promote tissue healing, such as cartilage repair, bone, and vascular remodeling, and involvement in inflammation regulation.^[10,15]^In recent years, PRP for KOA has become increasingly common in clinical practice and is a more promising treatment, but there are no clear conclusions about the role of PRP in KOA, and further high-quality studies are needed.

The wealth of contemporary scholarship and the increasing number of publications in the literature have contributed to making it increasingly difficult for professionals to keep up with the latest findings. While there is a constant flow of reviews analyzing current hot topics, some meaningful information, such as numerical growth trends, contributions of authors, institutions, and countries, and predictions of future research hotspots are often difficult to present.^[16]^ That is why bibliometrics comes into play. Bibliometrics is a tool to study trends and hotspots in a field by evaluating published articles through mathematical and statistical methods.^[17]^ The software Citespace developed by Prof Chen Chaomei allows visual analysis of authors, countries, institutions, cited documents, cited authors, and keywords and has been widely used for bibliometric analysis.^[18,19]^ A review of the literature has not revealed a bibliometric analysis of PRP on KOA treatment. Therefore, we conducted this study so as to uncover the state of research and provide prospects for future developments in the field.

## 2. Methods

### 2.1. Data collection

The data in the study were downloaded from the web of science Core Collection on July 18, 2022, and analyzed by searching for “Platelet-Rich Plasma” and “Osteoarthritis, Knee “. There were no restrictions on the type, language, or region of the literature, and the period of publication spanned from 2011 to 2021. A total of 988 publications were retrieved and exported as plain text files, with “Full record with cited references” selected as the export content. A total of 988 valid documents were obtained as a dataset for further analysis. Table 1 shows the specific search methods and the search results.

**Table 1:** The topic search query.

Set	Results	Search query
#1	146,28	TS = (PRP OR Platelet-rich plasma OR platelet-rich plasma OR platelet rich plasma OR platelet enriched plasma)
#2	35,531	TS = (Osteoarthritis, Knee OR Knees Osteoarthritis OR Knee Osteoarthritis OR Osteoarthritis, Knees OR Osteoarthritis of Knees OR Osteoarthritis of knee)
#3	988	#1 AND #2

PRP = platelet-rich plasma.

### 2.2. Data analysis

The data were imported into Citespace and 867 unique records were found after duplicates were removed. A bibliometric analysis was performed to understand hot spots and trends in publications, and the software presents annual publication counts, published journals, authors, countries, and institutions. By using nodes for correlation analysis, such as “references, authors, and institutions” can be selected in turn for visual analysis.^[18]^ The parameters of CiteSpace are set as follows: the time slice is from 2011 to 2021, “Years Per Slice” is selected as “1,” “Selection criteria” is set to the first “50,” and “pathfinder, pruning sliced networks” is selected for “Pruning” at the bottom of the Citespace interface. The rest of the options have no special modifications. The visual analysis consists mainly of nodes and links. Each node in the map represents an element, such as author, keyword, institution, etc. The size of the node indicates the frequency of co-occurrence or citation, while the different colors of the nodes indicate different years, with different colored circles indicating 2011 to 2021. In addition, links between nodes indicate collaboration or co-occurrence, or co-citation relationships. The purple circle represents centrality, and nodes with high centrality and high frequency are usually considered key points in the field.^[16,20]^

## 3. Results and discussion

### 3.1. Number of annual publications

**Figure 1.**
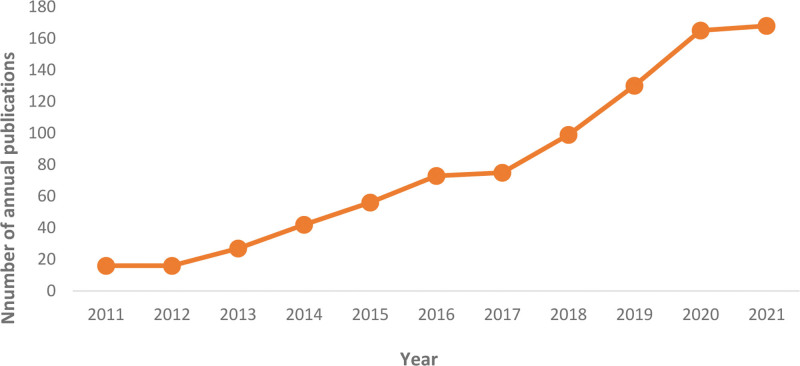
The annual number of publications between 2011 and 2021.

The analysis by Citespace revealed a total of 867 publications between 2011 and 2021, maintaining a certain number of publications each year and an overall upward trend, as shown in Figure 1. The figure shows that the least number of publications in the last decade was in 2011 and the highest in 2021. The growth was stable from 2011 to 2017, and after 2018 all maintained a high number of publications, showing a significant increase over the previous. It indicates that research on PRP for knee osteoarthritis has received more attention in the last 3 years with rich output.

### 3.2. Authors and cited authors

**Figure 2.**
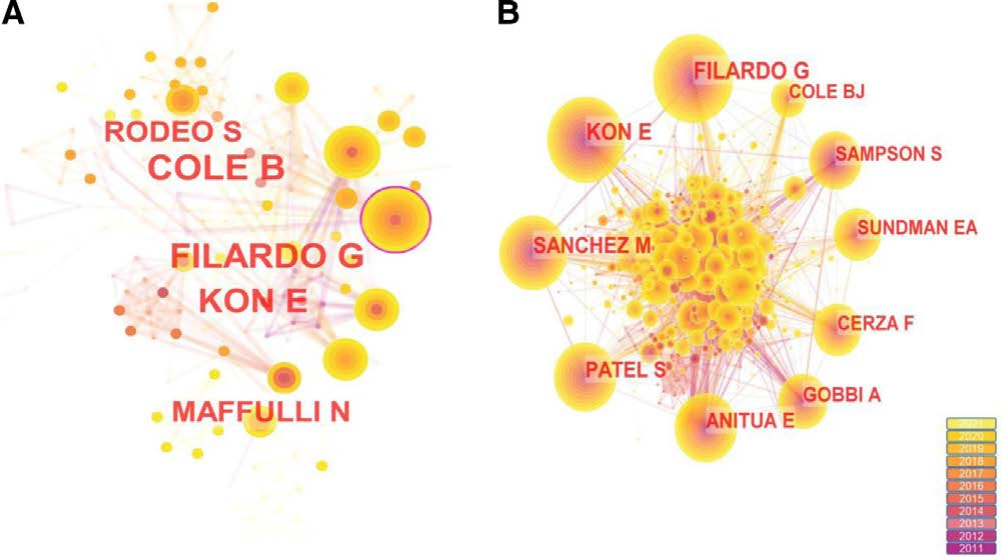
(**a**) Map of authors associated with PRP treatment KOA from 2011 to 2021. (**b**) Map of authors of subject-related citations from 2011 to 2021. KOA = knee osteoarthritis, PRP = platelet-rich plasma.

A total of 867 publications were analyzed, generating 390 nodes and 650 links, indicating that there were 390 authors in 867 publications, with closer collaboration among individual authors and mutual learning of scholarship (Fig. 2a). Table 2 shows the ranking of authors in terms of the number of publications, with the top 5 authors being Brian J. Cole, Giuseppe Filardo, Elizaveta Kon, Scott Rodeo, and Nicola Maffulli, respectively. Brian J. Cole is from the Department of Orthopaedics, Rush University Medical Center, Chicago, IL, and has made many contributions to KOA treatment has made many contributions. His and his team’s recent study concluded that intra-articular knee injections of PRP in KOA patients with clinical symptoms may improve pain and improve function in the short term. However, the effect of hyaluronic acid (HA) combined with PRP treatment warrants further study.^[21]^A recent Meta-analysis of 13 publications by Qi Zhang showed that PRP combined with HA treatment was not found to be superior to PRP treatment alone in terms of functional improvement and pain relief in KOA patients. However, in the incidence of adverse events, PRP combined with HA is usually safer than PRP injection alone.^[22]^ In a review by Brian J. Cole, it was shown that there are various devices used to centrifuge PRP and that differences in centrifugation speed and duration result in differences in platelet and leukocyte concentrations, and it was suggested that treating the knee with leukocyte-depleted PRP early in KOA could prevent or slow the onset of advanced KOA.^[23]^ The second-ranked author, Giuseppe Filardo, from the IRCCS Rizzoli Orthopaedic Institute in Bologna, Italy, showed in a 5-year double-blind, randomized controlled trial with Elizaveta Kon that both PRP and HA were effective in improving knee function, but follow-up failed to show that PRP had a clinical effect was better than that of HA.^[24]^ He also concluded that in most animal models, PRP attenuates the progression of cartilage damage and reduces the inflammatory response of the synovium, but high-level human trials are needed to demonstrate these positive results. This study was also coauthored by Mikel Sánchez.^[25]^ Although the clinical usefulness of PRP has been established by most authors, there is no consensus or standardization on some issues, such as the criteria of PRP preparation, centrifugation speed, and concentration, as well as the choice of indications and the frequency of injections.^[26]^

**Table 2:** Top 10 publication of author related to PRP therapy on KOA.

Rank	Publication	Author	Rank	Publication	Author
1	31	Brian J. Cole	6	14	Isabel Andia
2	28	Giuseppe Filardo	7	12	Mikel Sánchez
3	26	Elizaveta Kon	8	12	Berardo Di Matteo
4	16	Scott Rodeo	9	12	Maurilio Marcacci
5	15	Nicola Maffulli	10	11	Jorge A Chahla

KOA = knee osteoarthritis, PRP = platelet-rich plasma.

The map of cited authors is composed of 648 nodes and 1808 links (Fig. 2b), and the top 5 authors are Giuseppe Filardo, Elizaveta Kon, Mikel Sánchez, Sandeep Patel, and Eduardo Anitua. Elizaveta Kon has not only published more articles but has also been cited more frequently and has contributed significantly to the research in the field of PRP for KOA (Table 3). Mikel Sánchez has the 3rd highest number of citations. His earlier studies showed that PRP may regulate gene expression in cells such as chondrocytes and synovial cells, affect the anabolic microenvironment of the joint, maintain intra-articular stability and improve clinical symptoms.^[27]^ One of his retrospective studies showed that treatment with PRP injection before undergoing total knee arthroplasty between 2014 and 2019 delayed TKA by more than 1.5 years in 74.1% of patients. Survival analysis showed that 85.7% of KOA patients treated with PRP in 2014 and followed up until 2019 did not receive total knee arthroplasty during the 5-year follow-up. The severity of KOA did affect survival values, which were significantly higher in patients with non-severe KOA.^[28]^

**Table 3:** Top 5 cited author related to PRP therapy on KOA.

Rank	Frequency	Cited author
1	363	Giuseppe Filardo
2	323	Elizaveta Kon
3	245	Mikel Sánchez
4	222	Sandeep Patel
5	192	Eduardo Anitua

KOA = knee osteoarthritis, PRP = platelet-rich plasma.

### 3.3. Distribution of countries and institutions

**Figure 3.**
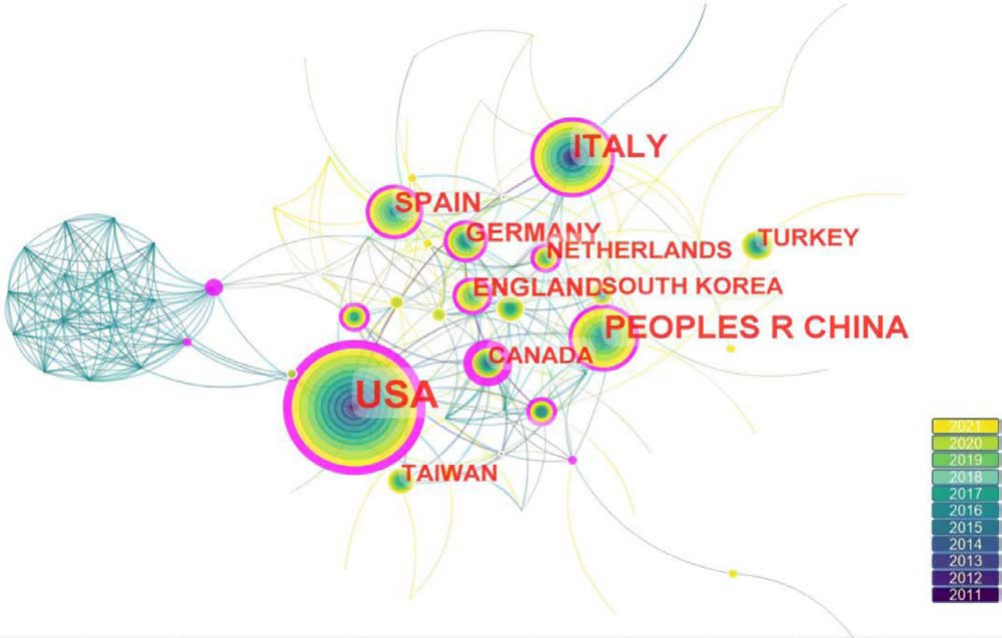
Country map of PRP treatment KOA from 2011 to 2021. KOA = knee osteoarthritis, PRP = platelet-rich plasma.

**Figure 4.**
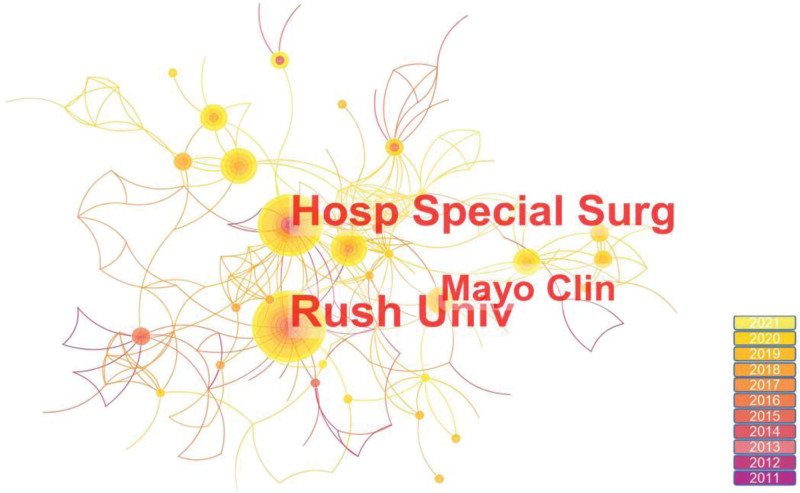
Institutional map of the RPR treatment KOA from 2011 to 2021. KOA = knee osteoarthritis.

The distribution map of countries or regions shows a total of 84 nodes and 309 links, indicating that 867 publications originated from 84 countries or regions (Fig. 3). The top 5 countries in the center in Table 4 are Argentina, the USA, Canada, Australia, and the Netherlands. The top 5 countries in terms of publications are the United States, China, Italy, Spain, and Germany. The United States not only has a high number of publications but also a high impact, indicating a high contribution to the field of PRP for KOA. China is second only to the USA in terms of the number of publications and has a low central position. Figure 4 shows the distribution of research institutions, with 344 nodes and 446 links. The top 5 institutions in terms of the number of publications are Rush Univ, Hosp Special Surg, Mayo Clin, IRCCS Ist Ortoped Rizzoli, and Queen Mary Univ London (Table 5). Hosp Special Surg had 32 publications, ranking 2nd, but its centrality (0.12) ranked first, so Hosp Special Surg has some influence in this field. The interconnection of institutions is not close, especially the research institutions that publish a lot of articles and have a large impact. As more and more research is conducted on hotspots, it is important to deepen cooperation and communication among various institutions and countries.

**Table 4:** The top 5 countries with the most publications and centrality.

Rank	Publications	Countries	Rank	Centrality	Countries
1	296	USA	1	0.4	Argentina
2	117	Peoples R China	2	0.27	USA
3	109	Italy	3	0.2	Italy
4	62	Spain	4	0.17	Canada
5	43	Germany	5	0.16	Netherlands

**Table 5:** The top 5 institutions with the most publications.

Rank	Publications	Institutions
1	36	Rush Univ
2	32	Hosp Special Surg
3	17	Mayo Clin
4	16	IRCCS Ist Ortoped Rizzoli
5	15	Queen Mary Univ London

### 3.4. Distribution of cited journals and cited references

**Figure 5.**
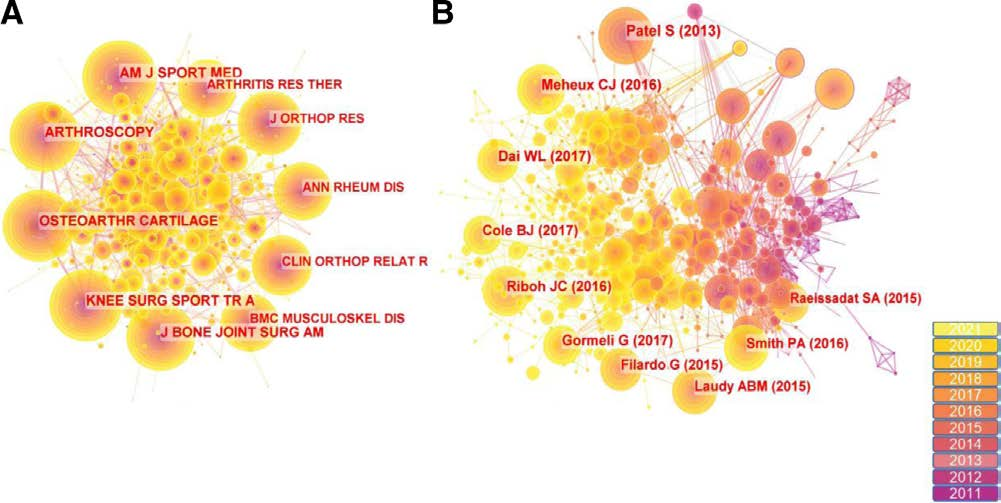
(**a**) Map of cited journal associated the RPR treatment KOA from 2011 to 2021. (**b**) Map of KOA cited references related to PRP from 2011 to 2021. KOA = knee osteoarthritis, PRP = platelet-rich plasma.

The cited journal distribution graph (Fig. 5a) consists of 597 nodes and 5273 links, reflecting the relationship graph of cited journals and co-citations. The analysis of the cited references graph (Fig. 5b) consists of 756 nodes and 3871 links. Tables 6 and 7 show the ranking of journals with high citation frequency and the citation frequency of references, it is interesting to note that 5 of the top 10 cited reference frequencies are articles from AM J SPORT MED and ARTHROSCOPY occupies 2. This indicates that these 2 journals are very important in the study of PRP for KOA, not only in terms of the number of articles published but also in terms of the content published, which is a hot topic in current research and guides both scientific and clinical work. In a double-blind, randomized, placebo-controlled trial, a short-term follow-up by Sandeep Patel showed the superiority of leukocyte-free PRP injections over the placebo group (saline injections) for the treatment of KOA and noted that no significant difference was seen in the short-term effect of 1 versus 2 PRP injections, but the difference became more pronounced with time.^[29]^ Carlos J Meheux concluded that patients with KOA who received PRP injections had significant symptomatic improvement over 12 months, but there was limited evidence comparing the effect of leukocyte-rich PRP (LR-PRP) with that of leukocyte-poor PRP.^[30]^ Wen-Li Dai, in a meta-analysis of randomized controlled trials, similarly showed that PRP injections were more effective than saline injections for KOA and noted that compared with HA and saline, PRP does not increase the risk of adverse events.^[31]^ A recent Meta-analysis of 20 randomized controlled trials and 3 prospective comparative studies showed no significant differences found between leukocyte-poor PRP (LP-PRP) and LR-PRP in terms of efficacy and local adverse effects in KOA patients. However, the surface under the cumulative ranking (SUCRA) showed that LP-PRP was superior to LR-PRP during follow-up when comparing all outcome indicators.^[32]^ In a randomized, double-blind, multicenter study, pure platelet-rich plasma (P-PRP) was shown to be superior to saline in the treatment of KOA. P-PRP was effective in relieving symptoms for at least 24 months, and the safety profile of P-PRP and saline was comparable.^[33]^

**Table 6:** Top 5 cited journals related to PRP therapy on KOA.

Rank	Frequency	Cited journals
1	714	Am J Sport Med
2	668	Arthroscopy
3	640	Osteoarthr Cartilage
4	601	Knee Surg Sport Tr A
5	520	J Bone Joint Surg Am

KOA = knee osteoarthritis, PRP = platelet-rich plasma.

**Table 7:** Top 10 cited references related to PRP therapy on KOA.

Rank	Frequency	Author and publication Year	Journals
1	118	Patel S, 2013	Am J Sport Med
2	115	Meheux CJ, 2016	Arthroscopy
3	104	Dai WL, 2017	Arthroscopy
4	97	Cole BJ, 2017	Am J Sport Med
5	92	Riboh JC, 2016	Am J Sport Med
6	92	Gormeli G, 2017	Knee Surg Sport Tr A
7	90	Filardo G, 2015	Am J Sport Med
8	89	Laudy ABM, 2015	Brit J Sport Med
9	87	Smith PA, 2016	Am J Sport Med
10	77	Raeissadat SA, 2015	Clin Med Insights-AR

KOA = knee osteoarthritis, PRP = platelet-rich plasma.

### 3.5. Analysis of keywords

**Figure 6.**
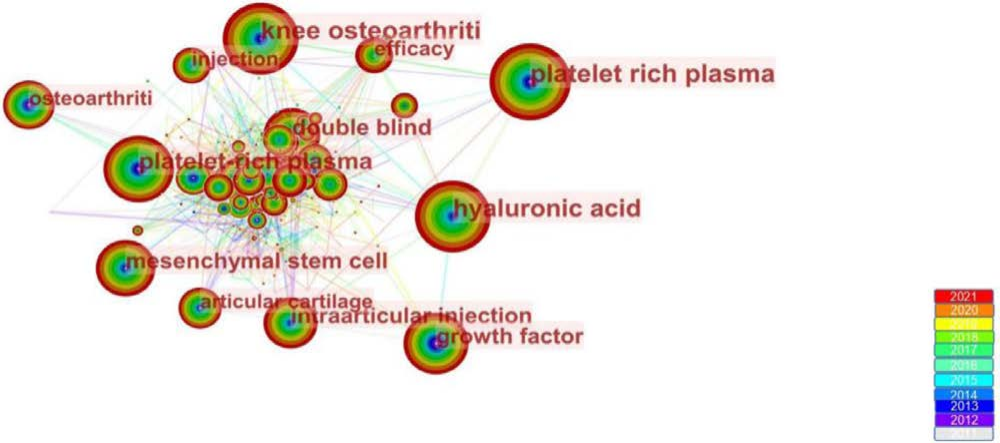
Map of keywords occurrence related to PRP for KOA from 2011 to 2021. 2021. KOA = knee osteoarthritis, PRP = platelet-rich plasma.

The high-frequency keywords of the summarized articles allow the analysis of research hotspots and emerging trends. The co-occurrence analysis graph of keywords consisted of 458 nodes and 3307 links. The more popular ones found according to frequency were platelet-rich plasma (415), knee osteoarthritis (397), hyaluronic acid (350), double-blind (213), and mesenchymal stem cells (197) (Fig. 6). HA or PRP is commonly used in clinical practice to treat KOA and is therefore a hot topic of scholarly interest. In a randomized double-blind trial, both platelet-rich plasma and hyaluronic acid were shown to be effective in the treatment of KOA and remained stable for up to 18 months after injection.^[34]^ Mesenchymal stem cells (MSCs) are generally considered to have a high osteogenic potential^[35]^ and have shown promising results in repairing damaged tissues in various degenerative diseases. MSCs have the homing ability, they reach the site of injury and differentiate directly into damaged tissues as well as secrete chemokines, cytokines, and growth factors that contribute to tissue regeneration.^[36–39]^ The combination of PRP and MSCs appears to be more beneficial for bone healing than PRP alone.^[40,41]^According to Meta-analysis, at 6-month follow-up, adipose mesenchymal stem cells provided better pain relief than PRP, HA, and saline, and LP-PRP was most effective for functional improvement. At the 12-month follow-up, both adipose mesenchymal stem cells and LP-PRP provided clinical pain relief, and knee function improved better with LP-PRP.^[42]^ In addition, studies have shown that bone marrow aspirate concentrate, which contains a relatively low percentage of MSCs, is not statistically different in terms of pain relief, functional improvement, and occurrence of adverse events compared to PRP in patients with KOA treated by injection.^[43,44]^

**Figure 7.**
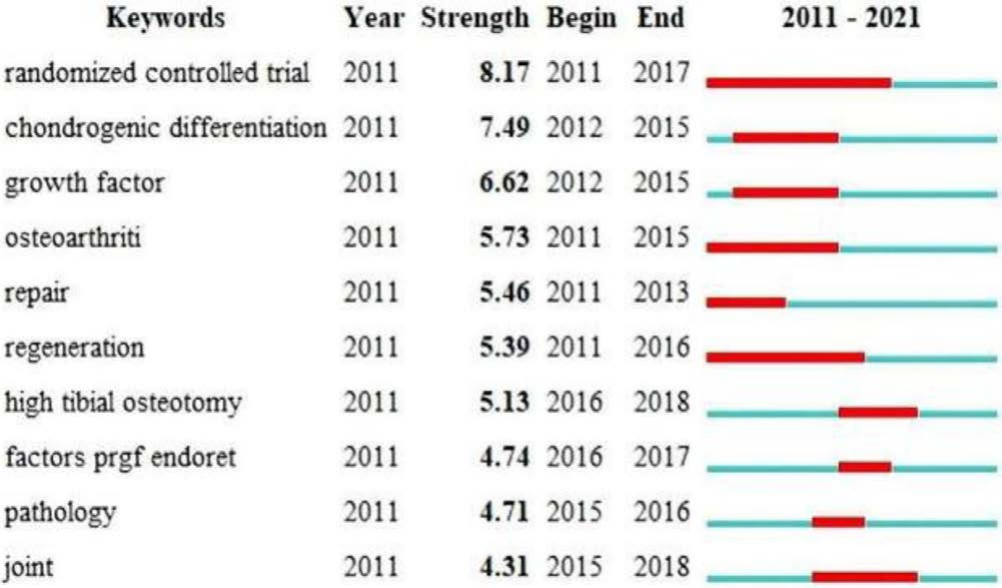
Top 10 keywords with the strongest citation bursts.

Figure 7 shows the top 10 strongest keyword bursts from 2011 to 2021. a randomized controlled trial is the most important study method to evaluate PRP for KOA. the Chondrogenic differentiation burst appeared in 2012 and ended in 2015 in the second place. Studies have shown that PRP may increase chondrocyte and mesenchymal stem cell proliferation, proteoglycan, and type II collagen deposition. It was also found that PRP increased the cell viability of chondrocytes, promoted migration and cartilage differentiation of MSCs, and inhibited the effects of catabolic cytokines.^[45]^

**Figure 8.**
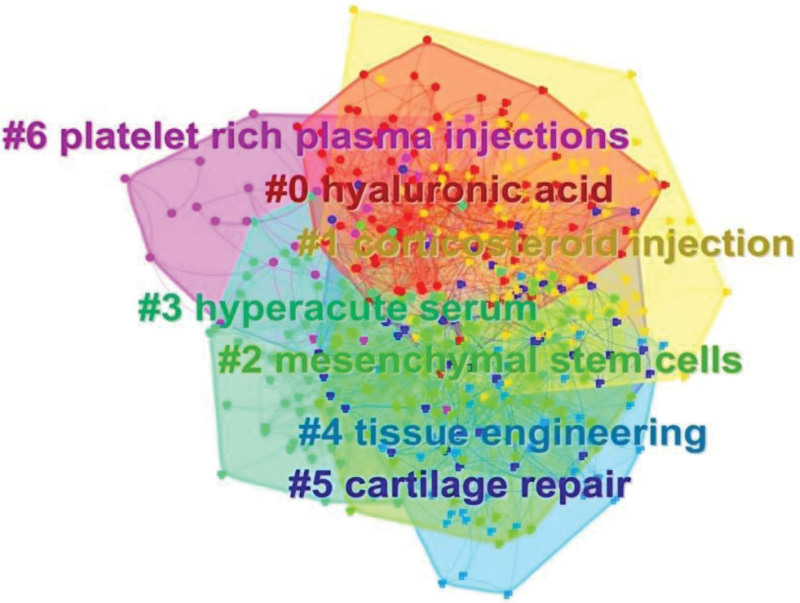
The clustering of keywords.

Figure 8 Cluster analysis on the basis of keyword co-occurrence to obtain the keyword co-occurrence clustering map of PRP therapy KOA. Clustering analysis is the process of grouping data without categorical information by certain methods according to the degree of similarity as a way to understand the basic knowledge structure of the domain. The keyword co-occurrence network of PRP Therapy KOA forms a total of 6 clusters. Each color block represents a cluster, and the nodes within the color block belong to that cluster. Cluster labels are usually extracted from titles, keywords, and abstracts using certain algorithms. The cluster number is inversely proportional to the cluster size, and the largest cluster is labeled with #0, and so on. To further analyze the relevant knowledge structure, the clusters are listed in Table 8 below.

**Table 8:** Details of keyword clustering.

Cluster ID	Size	Years	Clustering labels
0	93	2015	Hyaluronic acid
1	88	2015	Corticosteroid injection
2	81	2016	Mesenchymal stem cell
3	79	2016	Hyperacute serum
4	47	2015	Tissue engineering
5	45	2015	Cartilage repair
6	25	2019	Platelet rich plasma injections

## 4. Limitation

There are some limitations to the study. We searched the web of science for literature related to the topic, but because of the large amount of data, we could not guarantee that all the literature was closely related to the topic. Secondly, other databases were not searched and analyzed, such as China National Knowledge Infrastructure. Finally, the limitation of the search is that it is from 2011 to 2021, and the literature before 2011 may be at a higher citation frequency, but it cannot be analyzed as a co-occurrence. Recent breakthrough research advances were likewise not available for visual analysis.

## 5. Conclusion

With the analysis of a decade of literature studies, the research on PRP for KOA is in a good stage of development. Not only does the United States have the highest number of publications, but also the influential journals and institutions are from the United States. There is a lack of communication between different countries and institutions. The hot spot of contemporary research focuses on the effect of PRP on KOA, but there is a lack of high-level, multicenter, and large-sample studies. There is no standardized consensus on the preparation and composition of PRP. In addition in vivo experiments, repair of cartilage and other basic studies can also represent the frontier of research. In conclusion, bibliometric analysis can provide a quick understanding of the countries, institutions, authors, journals, and research hotspots in the field to help 1’s research direction.

## Acknowledgments

Thanks to Professor Chaomei Chen and his team for developing Citespace and making it available for free.

## Author contributions

**Conceptualization:** Yubo Cui.

**Data curation:** Yubo Cui, Liqiong Lin, Zhiwei Wang, Kai Wang, Lili Xiao, Wentao Lin.

**Investigation:** Liqiong Lin, Zhiwei Wang, Kai Wang, Lili Xiao, Wentao Lin.

**Methodology:** Wentao Lin, Yiyuan Zhang.

**Supervision:** Yiyuan Zhang.

**Visualization:** Yubo Cui.

**Writing – original draft:** Yubo Cui.

**Writing – review & editing:** Yiyuan Zhang.
